# DNA Barcodes of *Mansonia* (*Mansonia*) Blanchard, 1901 (Diptera, Culicidae)

**DOI:** 10.3390/genes14061127

**Published:** 2023-05-23

**Authors:** Jandui Almeida Amorim, Tatiane Marques Porangaba de Oliveira, Ivy Luizi Rodrigues de Sá, Taires Peniche da Silva, Maria Anice Mureb Sallum

**Affiliations:** 1 Departamento de Epidemiologia, Faculdade de Saúde Pública, Universidade de São Paulo, São Paulo 01246-904, SP, Brazil; porangaba@usp.br (T.M.P.d.O.); ivyluizisa@gmail.com (I.L.R.d.S.); masallum@usp.br (M.A.M.S.); 2 Departamento de Ciências e Matemática, Instituto Federal de Educação, Ciência e Tecnologia de São Paulo, São Paulo 01109-010, SP, Brazil; 3 Laboratório de Entomologia Médica, Instituto de Pesquisas Científicas e Tecnológicas do Estado do Amapá, Macapá 68903-419, AP, Brazil; tairespeniche@gmail.com

**Keywords:** cytochrome c oxidase subunit I, molecular taxonomy, species complexes, mosquitoes, disease vectors, Atlantic Forest, Amazon Forest

## Abstract

Females of the genus *Mansonia* feed on the blood of humans, livestock, and other vertebrates to develop their eggs. The females’ biting behavior may cause severe disturbance to blood hosts, with a negative impact on public health and economics. Certain species have been identified as potential or effective disease vectors. The accurate species identification of field-collected specimens is of paramount importance for the success of monitoring and control strategies. *Mansonia* (*Mansonia*) morphological species boundaries are blurred by patterns of intraspecific heteromorphism and interspecific isomorphism. DNA barcodes can help to solve taxonomic controversies, especially if combined with other molecular tools. We used cytochrome c oxidase subunit I (*COI*) gene 5′ end (DNA barcode) sequences to identify 327 field-collected specimens of *Mansonia* (*Mansonia*) spp. The sampling encompassed males and females collected from three Brazilian regions and previously assigned to species based on their morphological characteristics. Eleven GenBank and BOLD sequences were added to the DNA barcode analyses. Initial morphospecies assignments were mostly corroborated by the results of five clustering methods based on Kimura two-parameter distance and maximum likelihood phylogeny. Five to eight molecular operational taxonomic units may represent taxonomically unknown species. The first DNA barcode records for *Mansonia fonsecai*, *Mansonia iguassuensis*, and *Mansonia pseudotitillans* are presented.

## 1. Introduction

Some *Mansonia* species have been implicated as vectors of filariasis in Asia [1,2], Africa [3], and the Americas [4]. Many of these species were found to be naturally infected by arboviruses that can infect humans, causing diseases such as chikungunya [5], Zika fever [6], dengue fever [7], West Nile fever [8], Japanese encephalitis [9,10,11,12], Eastern equine encephalitis [13,14], Saint Louis encephalitis [15,16,17], and Venezuelan equine encephalitis [18,19], among others [5]. The subgenus *Mansonia* Blanchard, 1901 is widely distributed across the Americas, mainly in areas across the neotropical region [20]. *Mansonia* (*Mansonia*) *dyari* Belkin, Heinemann & Page, 1970 and *Mansonia* (*Mansonia*) *titillans* (Walker, 1848) are found from the southern United States to Central and South America, including the Caribbean islands [21]. *Mansonia titillans* and *Mansonia* (*Mansonia*) *indubitans* Dyar & Shannon, 1925 are vectors of Venezuelan equine encephalitis virus in Peru [22] and Venezuela [23]. Some species classified in the subgenus *Mansonia* are vectors of the Oriboca and Bussuquara viruses and likely of *Wulchereria brancrofti* Cobbold, 1877 in South America [5].

In addition, *Mansonia* mosquitoes can become disturbing pests for humans and livestock in areas where their populations reach high densities, given the voracity of hematophagous females [24]. Thus, monitoring populations of *Mansonia* species is necessary wherever these mosquitoes may pose a risk to human and animal health.

For the effective design of mosquito surveillance and control programs, it is essential to identify the target species accurately [25]. However, the small size of the specimens, frequent injuries during field collection, inadequate storage, and the growing shortage of well-trained professionals are among the obstacles to morphology-based species identification [26]. Added to these obstacles is the fact that several distinct species belong to morphologically similar complexes. In these complexes, both isomorphic and polymorphic taxa complicate species identification [26], for example, as for *Culex pipiens* [27] and *Anopheles gambiae* complexes [28]. Using molecular markers in taxonomy not only contributes to resolving morphology-based species delimitation impasses, but also serves to test species hypotheses, even if they have already been validated [29].

Since the publication of a seminal article by Hebert et al. [30], the 5′ end fragment of 658 base pairs of cytochrome c oxidase subunit I (*COI*) mitochondrial gene has been increasingly used as a DNA barcode for species identification, including mosquito disease vectors [26,31,32]. This molecular marker has allowed advances in exploring the genetic diversity of the mosquito fauna of Brazil [33,34,35,36,37,38], Argentina [33], Colombia [17,39,40], Ecuador [41], French Guiana [42], Mexico [43,44], Canada [45], the United Kingdom [46], Belgium [47], Sweden [48], Malawi [49], Saudi Arabia [50], Iran [51], Pakistan [52], India [53], Sri Lanka [54], Singapore [55], Turkey [56], China [57], Japan [58], and Australia [59].

The identification of mosquito species using *COI* sequences is usually in line with that based on morphology at or below genus level, but the DNA barcode region may not be adequate to identify cryptic mosquito species [26]. However, even when the *COI* gene does not clearly differentiate closely related species with recent divergence history, it demonstrates lineages that are better separated when additional nuclear markers are employed [60,61]. The *COI* sequences are relatively easy to obtain and abundantly available for a wide variety of taxa in publicly accessible databases such as GenBank and Barcode of Life Database (BOLD). This makes the DNA barcodes interesting as a molecular tool to verify a potential complex of morphologically similar species [42,43,44,57,59,62].

Bibliographic records show some disagreement regarding the taxonomy of the species in the subgenus *Mansonia* [63,64,65,66,67,68]. This can be explained by gaps in the morphological, biological, and ecological knowledge of most species. The immature stages and adult females of *Mansonia* (*Mansonia*) *pessoai* (Barreto & Coutinho, 1944), *Mansonia* (*Mansonia*) *cerqueirai* (Barreto & Coutinho, 1944), and *Mansonia* (*Mansonia*) *chagasi* (da Costa Lima, 1935) are unknown. The last taxonomic changes in the group were the revalidation of *Mansonia* (*Mansonia*) *fonsecai* (Pinto, 1932) [68]—previously in the synonymy of *Ma*. *indubitans*—and description of *Mansonia* (*Mansonia*) *iguassuensis* Barbosa, Navarro-Silva & Sallum, 2007 [69].

The hypothesis that there are species complexes in the subgenus *Mansonia* is not a novelty [66]. In such cases like, DNA barcodes have been employed to reveal and identify species that are grouped into morphologically similar taxa [26]. However, the results of the DNA barcode region comparisons are more robust and accurate when there is a taxon-specific sequence library [70]. In this investigation, we employed field-collected *Mansonia* (*Mansonia*) spp. specimens preliminarily identified at the species level using morphological characteristics. Subsequently, samples of each species, males, and females, and those with noticeable morphological variations, were separated for *COI* sequencing. The availability of *COI* sequences generated for the present study will improve the identification of *Mansonia* (*Mansonia*) spp. using DNA barcodes.

## 2. Materials and Methods

### 2.1. Mosquito Sampling

**Figure 1:**
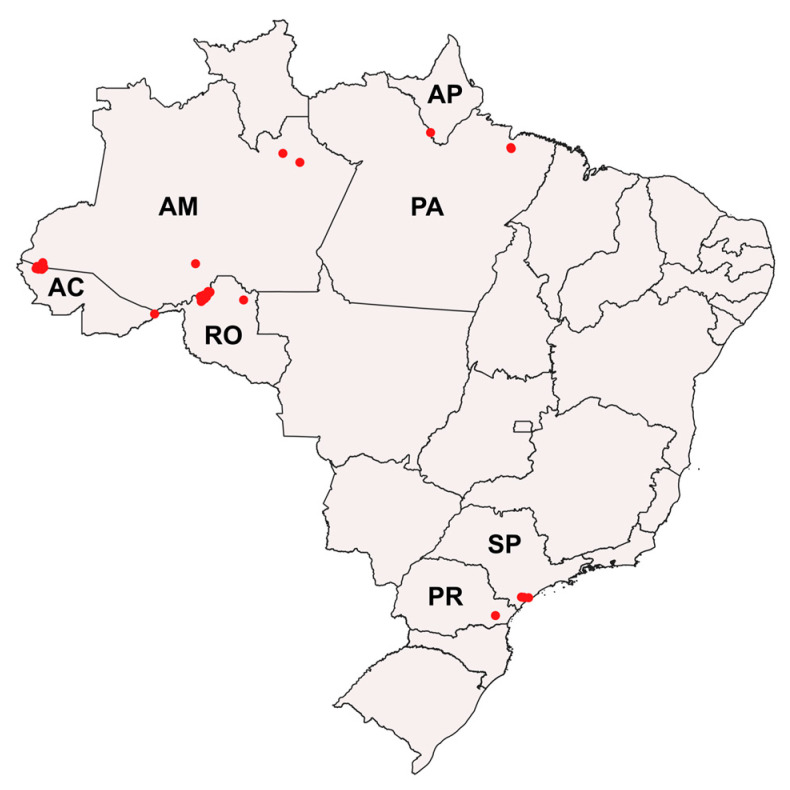
Distribution of *Mansonia* (*Mansonia*) spp. collection areas in the states of Rondônia (RO), Acre (AC), Amazonas (AM), Amapá (AP), Pará (PA), São Paulo (SP), and Paraná (PR), Brazil. Red points correspond to the specimen collection sites.

Adults and immatures of *Mansonia* species were collected from rural and urban areas in the Brazilian states of São Paulo (SP), Paraná (PR), Rondônia (RO), Acre (AC), Amazonas (AM), Amapá (AP), and Pará (PA), from April 2015 to August 2020 (Figure 1; see Supplementary Table S1 for geographic coordinates). Adults were collected from AC, AM, AP, and RO using automatic CDC light traps (CDC-LT), with manual electric catchers (EC) resting on the vegetation in early morning, human landing catch overnight, and the barrier screen sampling method (BSS) [71]. The CDC-LT and BSS collections were carried out from 6 p.m. to 6 a.m. Males and females were immediately euthanized with ethyl acetate vapor and placed in plastic vials containing silica gel for transport and storage until morphological identification. In the Laboratory of Entomology in Public Health-Culicidae systematics, of the Faculty of Public Health of the University of São Paulo, São Paulo, Brazil, specimens that were morphologically identified at the species level were separated for generation of DNA barcode sequences.

In addition to adults, larvae, and pupae of *Mansonia* spp. were sampled from streams, backwaters, ponds, and flooded areas in the Brazilian states of São Paulo, Paraná, Pará, and Rondônia. Each collection site was sampled for three to four hours. Individuals of *Eichhornia crassipes* (Mart.) Solms (Commelinales: Pontederiaceae) and *Pistia stratiotes* L. (Alismatales: Araceae) were chosen randomly at different points in the aquatic system and searched for larvae and pupae attached to the roots. The plants were taken from the water and their roots were vigorously shaken in a 20-L bucket containing sieved water from the habitat. The water was visually searched for third- and fourth-instar larvae and pupae. Once found, the specimens were separated using a 3 mL plastic pipette. The immatures were transferred and kept alive in sterile Whirl-Pak^®^ sample bags containing water from the habitat previously sieved to remove any predators. In addition, one or more *P*. *stratiotes* plants were included in the sample bag as substrate for larvae and pupae. These plants were the only species used for the transportation of immature culicids and maintenance under laboratory conditions because they fitted into the sample bag and into the laboratory container, unlike *E. crassipes*. The sample bags were transported to the Laboratory of Entomology in Public Health-Culicidae, Faculty of Public Health of the University of São Paulo, São Paulo, Brazil. Field-collected larvae and pupae were kept individually in colorless 500 mL plastic vials with approximately 300 mL of sieved water from the habitats. Each container was identified according to the sample site and covered with a piece of plastic mesh attached to its top to prevent any disturbances to the developing immature mosquitoes. Adults that emerged in the laboratory were kept alive for at least 12 h before they were euthanized, identified at the species level, and stored individually in small plastic vials in silica gel just as the field-collected adults.

### 2.2. DNA Extraction

Genomic DNA was extracted from one or two legs of each mosquito specimen. For each extraction, the legs were macerated in 10 μL of 0.9% NaCl with an autoclaved pistil and then 20 μL of 5% Chelex-100 was added. The solution was vortexed and incubated at 99 °C for 10 min. After centrifugation at 13,000 rpm at 25 °C for 5 min, the supernatant was recovered, and an aliquot was frozen at −20 °C for PCR amplification of the target *COI* gene region. The remaining DNA was stored at −70 °C in the frozen entomological collection of the Faculty of Public Health, São Paulo, Brazil.

### 2.3. DNA Amplification

The primers LCO1490 5′-GGTCAACAAATCATAAAGATATTGG-3′ and HCO2198 5′-TAAACTTCAGGGTGACCAAAAAATCA-3′ [72] were used to amplify the *COI* gene barcode region, according to Bourke et al. [35]. Each reaction was performed in a total volume of 25 μL containing 2 μL of DNA, 1× PCR buffer (Invitrogen), 1.5 mM MgCl2 (Invitrogen), 0.2 mM of each dNTP (Amresco), 0.1 μM of each primer, and 0.625 U of Taq Platinum polymerase (Invitrogen), and the remaining volume consisted of ultrapure water. The PCR thermal conditions consisted of 94 °C for 3 min, five cycles of 94 °C for 30 s, 45 °C for 90 s, 68 °C for 60 s, followed by 35 cycles of 94 °C for 30 s, 51 °C for 30 s, 68 °C for 60 s, and a final extension at 68 °C for 10 min. PCR products were purified by PEG precipitation (20% polyethylene glycol 8000/2.5 M NaCl).

### 2.4. Sequencing

Sequencing reactions were performed in both directions using a Big Dye Terminator cycle sequencing kit v3.1 (Applied Biosystems, Foster City, CA, USA), and the same set of PCR primers. Each sequencing reaction was carried in a total volume of 10 μL containing 1 μL of purified PCR product, 0.5 μL of Big Dye^®^ Terminator v3.1 Ready Reaction Mix (PE Applied Biosystems), 1× sequencing buffer (PE Applied Biosystems), and 3.6 pmol of F or R primer, and the remaining volume consisted of ultrapure water. The sequencing products were purified with Sephadex G50 columns (GE Healthcare) and analyzed in an Applied Biosystems 3130 DNA Analyser (PE Applied Biosystems, Warrington, UK). Sequences were edited using the Sequencher software v. 5.2.4 (Genes Codes Corporation, Ann Arbor, MI, USA) and the primer regions were removed.

### 2.5. Sample Identification and COI Database

All adult specimens were preliminarily identified at the species level using the identification keys of Barreto and Coutinho [64], Forattini [24], Barbosa [73], and Assumpção [74]. Male specimens had their identification checked by examining their genitalia mounted on microscope slides. The prior morphological identification of mosquitoes was then revised, considering the species geographic distribution records and the analysis of the *COI* gene sequences.

Searches were performed with the Basic Alignment Search Tool (BLAST), available at https://blast.ncbi.nlm.nih.gov/Blast.cgi (accessed on 8 April 2023), for all DNA barcode sequences to assess their similarities to those available from *Mansonia* species and to confirm that they were coding data, free of indels, nuclear mitochondrial DNA sequences (NUMTS), or stop codons. All sequences included in the analyses were 88.47–100% similar to other *Mansonia* (*Mansonia*) spp. sequences from GenBank ≥ 642 bp in length (Supplementary Table S2). Muscle was implemented in Mega X [75] for the alignment of nucleotide sequences. Under the reference of the *Mansonia* (*Mansonia*) *amazonensis* (Theobald, 1901) mitochondrion complete genome sequence available in GenBank (accession number MK575483), it was possible to verify that the 658 bp fragment of the *COI* gene corresponds to position 1509–2166. All 327 new sequences obtained for this study were deposited in GenBank with access numbers from OP785292 to OP785618 (Supplementary Table S1).

To reinforce the specific identity of the new DNA barcode sequences, 10 fragments of the *COI* gene from GenBank and one from BOLD were added to the dataset. GenBank sequences were generated from specimens identified as *Mansonia amazonensis* (NC044657 and MK575483), *Mansonia* (*Mansonia*) *flaveola* (Coquillett, 1906) (JX260065), *Ma. indubitans* (MN997669, MN997670, MN997671, and MN997672) and *Ma. titillans* (MN997665, MN997666, and MN997667). One *Ma. dyari* sequence was downloaded from BOLD (MOSN659-18.COI-5P). All 338 sequences were aligned using Muscle, as described above. As the *Ma. amazonensis* sequences obtained from GenBank corresponded to the complete mitochondrial genome, they were trimmed after alignment to match their length to that of the other sequences (658 base pairs).

A descriptive overview of the dataset was provided by MEGA X. The numbers of variable, conserved and parsimony-informative sites were determined. Three algorithms were applied for calculation of genetic distances: Kimura two-parameter (K2p) [76], Jukes-Cantor (J-C) [77], and Tamura three-parameter (T3p) [78]. As the results obtained from the three algorithms did not differ substantially, only K-2-p distances were used for the estimation of a neighbor-joining (NJ) tree–bootstrap method with 1000 replications and other distance-based cluster analyses (see Supplementary Tables S3 and S4 for J-C and T3p distances, respectively). In order to reduce the computation time spent on NJ analysis, the haplotype was identified using TCS version 1.21 [79] and multiple identical conspecific sequences were collapsed. Four sequences available in GenBank were used as outgroups: *Mansonia* (*Mansonioides*) *africana* (Theobald, 1901) (KU380402), *Coquillettidia (Rhynchotaenia) venezuelensis* (Theobald, 1912) (OP785619), *Coquillettidia* (*Coquillettidia*) *perturbans* (Walker, 1856) (JF867689), and *Psorophora* (*Janthinosoma*) *longipalpus* Randolph & O’Neill, 1944 (JX260114). The NJ tree was rooted in the *Ps. longipalpus* sequence using Figtree version 1.4. (http://tree.bio.ed.ac.uk/software/figtree/, accessed on 8 April 2023). The topology of the NJ tree supported the grouping of sequences according to their separation into lineages formed by terminal branches present in at least 99% of the bootstrap replication. These lineages were also referred to as molecular operational taxonomic units (MOTUs) [80]. The MOTUs that segregated specimens morphologically assigned to the same species were shown by “G (Group) I”, “G II” and, when necessary, also “G III” and “G IV” following the morphospecies name. Matrices of intra- and inter-MOTU genetic distances are provided in the results.

### 2.6. Cluster Analyses

Partition groups of all data were inferred from ranked distances using online versions of Automatic Barcode Gap Discovery (ABGD) [81] and Assemble Species by Automatic Partitioning (ASAP) [82]. The settings applied to the ABGD run were P_min_ = 0.001, P_max_ = 0.1, steps = 10, X = 1.5, and Nb bins = 20. The K2p substitution model was selected for both ABGD and ASAP distance-based methods. Pairwise distances were computed using the Refined Single Linkage (RESL) algorithm implemented on the BOLD Systems workbench to designate MOTUs, as described by Ratnasingham and Hebert [83].

Putative species groups were alternatively inferred based on the number of substitutions using the multi-rate Poisson tree processes (mPTP) version 0.2.4 [84]. A Markov Chain Monte Carlo (MCMC) of 10 million generations, sampling every 10,000 generations, was conducted in four independent runs supported by the mPTP model. Samples generated in the first step of MCMC and branches with lengths smaller than or equal to 0.0029103938 were discarded. As for the outgroup, a 658 bp *COI* sequence of *Ps. longipalpus* from GenBank (JX260114) was selected. As input for mPTP, we used a maximum likelihood (ML) best tree result created using RAxML version 8.2.12 [85] with rapid bootstrapping (-f a), random seed (-x 12345), and the GTRCATI substitution model.

## 3. Results

### 3.1. Sample Identification and COI Database

The prior morphological identification attributed 209 females and 118 male *Mansonia* (*Mansonia*) specimens to eight species: *Ma. amazonensis* (n = 22), *Ma. flaveola* (n = 13), *Ma. fonsecai* (n = 28), *Ma. humeralis* (n = 64), *Ma. iguassuensis* (n = 5), *Ma. indubitans* (n = 79), *Mansonia* (*Mansonia*) *pseudotitillans* (Theobald, 1901) (n = 3), and *Ma. titillans* (n = 113) (see Supplementary Table S1 for more details on developmental stage, sex, collection date, site, and method concerning each specimen). As the identification of the specimens was essentially based on morphological characters—although their geographical origin was also considered—the taxa were henceforth treated as morphospecies. Considering the 658 bp DNA barcode sequences obtained from 327 analyzed specimens, 436 sites were conserved (66.26%), 212 were variable (33.74%), 205 were parsimony-informative (31.16%), and 17 were singleton sites (2.58%). Sequences were AT rich (average 38.6% T; 17.0% C; 29.3% A; and 15.1% G), especially at the third codon position (average 45.4% T, 7.7% C, 45.0% A and 1.9% G).

### 3.2. NJ and Distance Analyses

**Figure 2:**
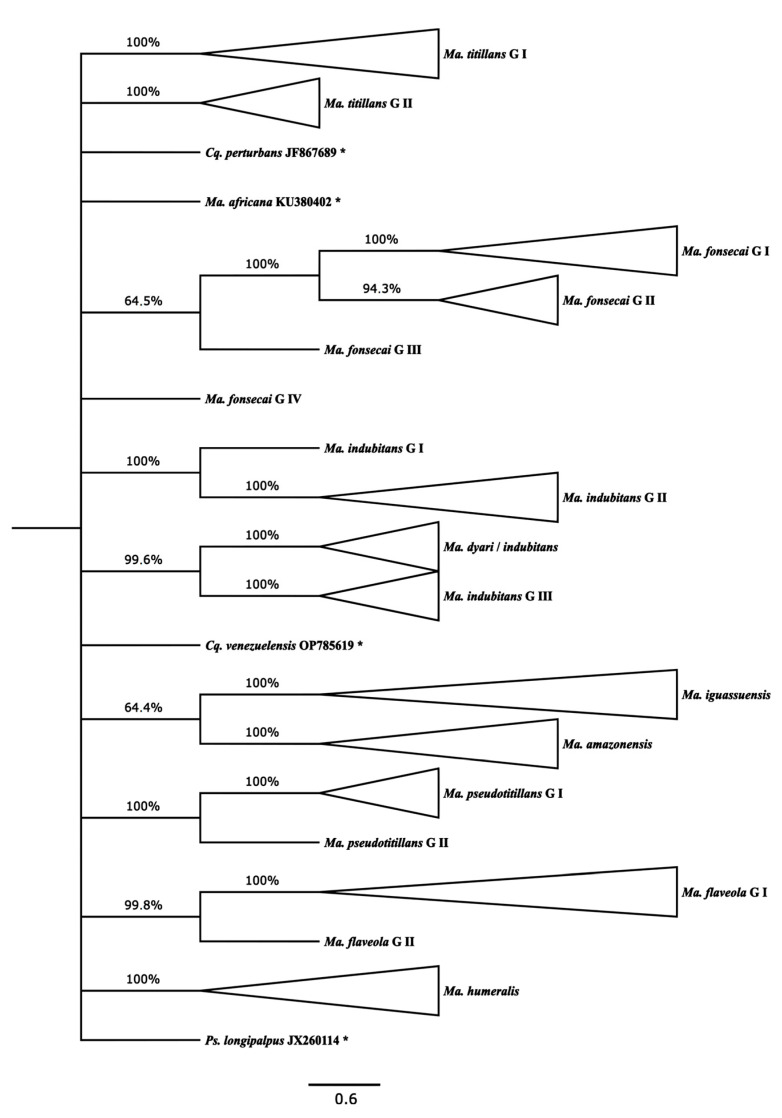
NJ topology estimated by MEGA X using K2p genetic distances (scale bar) with 124 *Mansonia* (*Mansonia*) spp. *COI* haplotype sequences, eight of which were extracted from Genbank and BOLD (summarized from Supplementary Figure S1). Only branches present in at least 60% of the bootstrap replicates are shown. The tree is rooted on the *Ps*. *longipalpus* sequence, which, as with those of the other outgroup members, is indicated by an asterisk. Bootstrap values (1000 replicates) are shown above the branches.

Figure 2 presents an NJ tree with collapsed terminal branches (see Supplementary Figure S1 for non-collapsed branches and Supplementary Table S1 for the correspondence of individual sequences with their respective haplotypes and MOTUs). The estimated NJ topology showed that the sequences of *Ma*. *flaveola*, *Ma*. *titillans*, and *Ma*. *pseudotitillans* were split into two distinct sibling lineages (MOTUs), namely, G I and G II for each morphospecies. Of the 14 sequences of *Ma*. *flaveola* analyzed, only one, extracted from a specimen from Puerto Rico, was segregated from others into *Ma. flaveola* G II, keeping the other 11 haplotypes, originating from the states of Rondônia and Acre, gathered into *Ma. flaveola* G I.

*Ma. titillans* G I gathered 30 haplotypes, representing specimens from the northern region of Brazil, the states of Acre, Rondônia, and Amapá. An additional 10 haplotypes, from the state of São Paulo, southeastern Brazil, and Rondônia, constituted *Ma. titillans* G II. Only sequences from Rondônia were included in *Ma. pseudotitillans* G I and *Ma. pseudotitillans* G II.

*Ma. fonsecai* and *Ma. indubitans* sequences were distributed among four MOTUs for each morphospecies. All *Ma. fonsecai* sequences were extracted from specimens collected in the state of Paraná, southern Brazil. *Ma. fonsecai* G I and *Ma*. *fonsecai* G II aggregated three and two different haplotypes, respectively. The other two remaining haplotypes for this morphospecies were segregated into two distinct MOTUs, *Ma*. *fonsecai* G III and *Ma*. *fonsecai* G IV, making it the most divergent among all four.

A single sequence of *Ma. indubitans,* extracted from a specimen collected in the state of Amazonas, was segregated from all the other sequences into *Ma. indubitans* G I. Its sister lineage, *Ma. indubitans* G II, included three other haplotypes from the states of Amazonas and Rondônia. *Ma. Indubitans* G III, the most divergent among the three MOTUs, assembled 15 haplotypes, seven of which included sequences from the type locality—in the state of Pará—mixed or not with other sequences from the states of Acre and Rondônia. The fourth MOTU included five haplotypes, four of them supposedly *Ma. indubitans* specimens from Colombia and another from a Puerto Rican specimen identified as *Ma. dyari.* Therefore, this last MOTU was named *Ma. dyari/indubitans*.

All *Ma. amazonensis*, *Ma. humeralis*, and *Ma. iguassuensis* sequences, represented by 17, 17, and 4 haplotypes, respectively, were grouped into conspecific distinct lineages defined as MOTUs. Those of *Ma. amazonensis* were generated from specimens collected in the states of Acre, Amazonas, and Rondônia. The origins of the *Ma. humeralis* specimens used in the study are in these same three states, in addition to São Paulo, the only state where *Ma. iguassuensis* was sampled. Finally, no outgroup sequences joined any lineage formed by those outgroups of *Mansonia* (*Mansonia*) spp.

Overall, the mean distance among all sequences was approximately 12.75% ± 0.92%. The average of the distances within the morphospecies groups were all >1% just for *Ma. amazonensis*, *Ma humeralis*, and *Ma. iguassuensis*. For the other morphospecies, such values ranged from 2.19% ± 0.46% (*Ma. pseudotitillans*) to 6.52% ± 0.65% (*Ma. fonsecai*). After progressively dividing the sequences into groups, corresponding to the lineages seen in the NJ tree topology, the intra-MOTU mean K2p distances were recalculated (Table 1).

At the maximum number of lineages after progressive partitions, *Ma. flaveola* G I showed the highest value among intra-MOTU mean distances, 0.83% ± 0.20%. This distance is less than half of the lowest one found by comparing the average of distances between different MOTUs, 2.15% ± 0.54%, involving *Ma. fonsecai* G I and *Ma. fonsecai* G II (Table 2; see Supplementary Table S5 for pairwise comparisons of all haplotype sequences). *Ma. flaveola* G I and *Ma. indubitans* G I were the most genetically divergent MOTUs (19.44% ± 1.95%).

### 3.3. Cluster Analyses

**Figure 3:**
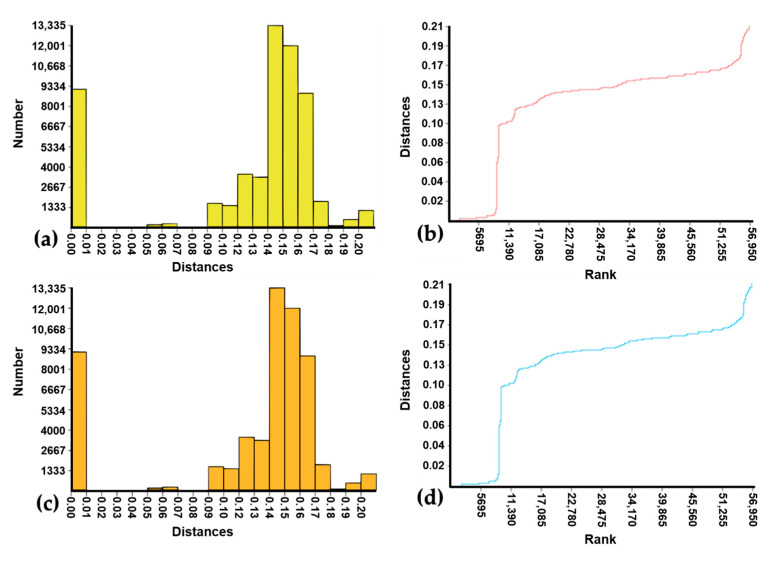
Histogram and rank of pairwise K2p distances generated by ABDG ((**a**) and (**b**), respectively) and ASAP ((**c**) and (**d**), respectively) with 338 *Mansonia* (*Mansonia*) spp. DNA barcode sequences (11 of them from GenBank and BOLD). The frequencies of the pairwise distances computed by ABDG and ASAP are shown by yellow columns (**a**) and orange columns (**c**), respectively; and ranked ordered pairwise distances computed by ABDG and ASAP are shown by red curve (**a**) and blue curve (**b**).

After including 10 sequences of *Ma. amazonensis*, *Ma. flaveola*, *Ma. indubitans*, and *Ma. titillans* from GenBank and one of *Ma. dyari* from BOLD, the automated clustering analyses were carried out with 338 individual sequences. This sequence set was partitioned into subsets (MOTUs) independently by ABGD, ASAP, RESL, and mPTP. The results were very congruent among the four algorithms and in relation to the lineages generated in the NJ analysis (Table 3). The ABDG and ASAP results showed some variations in the numbers of MOTUs, according to the computed prior limit to intraspecific diversity (*P*) [81] and threshold distance (*d_T_*) [82] values, respectively. Thus, Table 3 presents only the more biologically plausible results for *P* and *d_T_* values considered for these two algorithms (see Supplementary Table S6 for complete results). The initial partition of ABGD analysis found 13 MOTUs, independently of the *P* value, and the computed barcode gap [86] is illustrated by the histogram and the rank of distances (Figure 3, a and b, respectively). The recursive partition generated 14 MOTUs when P was 5.99%, 15 MOTUs when P was 3.59% or 2.15%, and repeatedly 16 MOTUs when P ranged from 0.17% to 1.29%. The barcode gap computed by ASAP did not differ from that of ABGD (Figure 3c,d). The ASAP algorithm segregated the sequences into 17 and 16 MOTUs when the *d_T_* values were 1.38% and 2.47%, respectively. The sequence partition with the lowest ASAP score—the best, according to Puillandre et al. [82]—distributed the sequences into 15 MOTUs according to *d_T_* = 3.50%. The same sequence clustering pattern was successively maintained by increasing the *d_T_* value to the limit of 10.09%.

The remaining two clustering methods, mPTP and RESL, generated 16 and 17 MOTUs, respectively. The RESL results are summarized in Table 4, where each MOTU is associated with a corresponding barcode index number (BIN) [83]. The sequence composition of the MOTUs generated by RESL mirrors was exactly that of the NJ analysis. The mean intra-MOTU K2p distances ranged from 0.00% to 0.83%. Only *Ma. flaveola* G I and *Ma. titillans* G I, the maximum intra-MOTU distances, exceeded 1%, but none of them reached 1.7%. The smallest inter-MOTU K2p distance occurred between *Ma. fonsecai* G I and *Ma. fonsecai* G II (1.83%). All other nearest neighbor (NN) distances ranged from 3.06% to 11.16%.

The results of all clustering methods generated a single MOTU for each of the three morphospecies, *Ma. amazonensis*, *Ma. humeralis*, and *Ma. iguassuensis*, identical to the NJ lineages. They split the *Ma. flaveola* and *Ma. titillans* sequences into two MOTUs for each morphospecies, also corresponding exactly to the composition of their respective G I and G II NJ lineages.

The sequences of some morphospecies clustered variably in ABDG and ASAP as the automated algorithm considered different genetic distance limits for the putative species.

Considering the delimitation of groups, those of the NJ lineages *Ma. fonsecai* G I and *Ma. fonsecai* G II were merged into a single MOTU in the results of ABGD (when *P* ≥ 2.15%), ASAP (when *d_T_* ranged from 2.47% to 10.09%), and mPTP. However, the splitting of this single sequence subset, generating those corresponding to lineages *Ma. fonsecai* G I and *Ma. fonsecai* G II, took place when ABGD’s *P* value ranged from 0.17% to 1.29%, ASAP’s *d_T_* = 1.38, and in the RESL results. The remaining *Ma. fonsecai* sequences invariably generated two MOTUs that were identical to the NJ lineages *Ma. fonsecai* G III and *Ma. fonsecai* G IV. In the same way, all the *Ma. pseudotitillans* sequences gathered into a single cluster in the ABGD and ASAP results, but for the latter, only if *d_T_* ≥ 3.50%. When ASAP’s *d_T_* ≤ 2.47%, the *Ma. pseudotitillans* sequences were divided into two MOTUs, corresponding to the NJ lineages *Ma. pseudotitillans* G I and *Ma. pseudotitillans* G II, as well as into mPTP and RESL outcomes. With just one exception, four MOTUs were generated in the segregation of *Ma. indubitans* sequences, faithfully reflecting the composition of the NJ lineages for this morphospecies, *Ma. indubitans* G I–G III and *Ma. dyari/indubitans*. This last MOTU gathered only the GenBank’s *Ma. indubitans* sequences and that of *Ma. dyari* from BOLD. However, when *P* = 5.99%, the ABDG analysis generated only two MOTUs, one of which aggregated the sequences of the NJ lineages *Ma. indubitans* G I and *Ma. indubitans* G II and all the remaining sequences of *Ma. indubitans* and *Ma. dyari*.

## 4. Discussion

Although the morphological identification of *Mansonia* mosquitoes did not prove to be complicated at the genus level, the same cannot be said about species identification. When identifying samples collected with CDC-LT, the difficulties become more prominent because of damage to specimens and loss of characters with taxonomic importance, such as bristles and scales. There is considerable uncertainty about the morphological identification of some specimens assigned to *Ma. titillans*, *Ma. indubitans*, and *Ma. fonsecai.* These species have often been mistaken for one another over the years, and to distinguish them, laborious and time-consuming preparation of the terminalia on microscope slides is needed [63,64,65,66,67,68].

When even after examining morphological, ecological, and geographic data, there is still doubt about the specific identification of specimens, incorporating molecular data into the analyses can help to elucidate species boundaries [87]. Despite the great efforts devoted to the construction of DNA barcode libraries for mosquito species of health interest [26,31,32], some groups remain somewhat neglected, with relatively few sequences available in publicly accessible databases, as is the case of *Mansonia* (*Mansonia*) spp.

We analyzed 327 new DNA barcode sequences extracted from mosquitoes of the subgenus *Mansonia* morphologically assigned to eight species (Supplementary Table S1). There was a notorious A + T nucleotide bias in these sequences, mainly at the 3rd codon position, in agreement with previous analyses of the *COI* gene sequences of other mosquito genera [33,45,57,59]. In fact, this seems to be a pattern concerning fragments of the mitochondrial genome of insects [88]. The proportions of conserved, variable, parsimony-informative and singleton sites were consistent with those of other previously published *Mansonia* (*Mansonia*) spp. DNA barcode sequences [38].

Neighbor-joining analysis based on K2p distances with 1000 replicates segregated the *Ma. amazonensis*, *Ma. humeralis*, and *Ma. iguassuensis* sequences into three well-supported conspecifics (100%) lineages treated here as MOTUs. Their intra-MOTU mean K2p distances ranged from 0.36% ± 0.18% to 0.54% ± 0.13% (Table 1), but the average of all pairwise distances between members of these three MOTUs and those of any other MOTUs was ≥10.39% ± 1.28% (Table 2). Congruently, the maximum K2p distances computed by RESL within MOTUs corresponding to *Ma. amazonensis*, *Ma. humeralis*, and *Ma. iguassuensis* ranged from 0.46% to 0.92%, while the NN distance ranged from 9.48% to 11.16% (Table 4).

*Ma. humeralis* sequences were extracted from specimens collected from both the Amazon (western, central, and eastern) and from the southeastern coast of Brazil, representing the genetic variability among geographically very distant populations. The results of the four clustering methods implemented, whether based on genetic distances or on the number of nucleotide substitutions, aggregated all these sequences into a single MOTU, corroborating those of the NJ analysis. All specimens from which the sequences in question were extracted showed golden scales symmetrically covering the two anterolateral areas of the scutum and erect scales in the basal portion of the anterior tibiae, typical characters of *Ma. humeralis* [64,89]. Considering the body of evidence presented here, the molecular identification of *Ma. humeralis* fully corresponded to that based on morphology.

The results of the molecular analyses for species delimitation confirmed the morphological identification of the specimens morphologically assigned to *Ma. amazonensis* and *Ma iguassuensis*. The loss of the golden scales that normally cover the entire scutum of *Ma. amazonensis* specimens can be mistaken for those of *Ma. wilsoni* (unfortunately, not sampled in our collections), especially in the case of females [75]. In turn, *Ma. iguassuensis* was described less than 20 years ago by Barbosa et al. [69], according to whom the species can be misidentified as *Ma. titillans*, *Ma. indubitans*, *Ma. wilsoni*, or *Ma. humeralis*. Therefore, the possibility of identifying *Ma. amazonensis* and *Ma iguassuensis* through DNA barcodes provides an optimistic perspective. Especially the identification of *Ma. iguassuensis* can be reassessed by using DNA barcode sequences, given its likely preservation in collections misidentified as morphologically similar species. It is fair to declare that there was only one circumstance in which the molecular and morphological identification of the *Ma. amazonensis* and *Ma. iguassuensis* specimens did not match. When ASAP based the partition on *d_T_* = 11.74%, a single MOTU gathered the sequences of these two species plus those of *Ma. fonsecai*, *Ma. indubitans*, *Ma. dyari*, and *Ma. titillans* (Supplementary Table S6). The morphological differences between some pairs of these six species [24,64,68,69] and the high score of the ASAP gap width (11.00) make this an unlikely outcome, which will therefore be neglected.

At the opposite extreme, ASAP *d_T_* (0.76%) was the only case in which the result of an analysis segregated *Ma. flaveola* sequences into four MOTUs instead of two, as output by the other analytical methods. According to maximum conspecific distances for various mosquito genera published in the last two decades, this distance threshold was very low to separate non-conspecific sequences [44,45,52,53,57,59]. Thus, it is not credible that the sequences morphologically assigned to *Ma. flaveola* could really represent four distinct species. When these sequences were analyzed all together, the computed average of pairwise distances was 2.32% ± 0.29%. This value is within the range of those proposed by Hebert et al. [30] as thresholds of intraspecific divergence to delimit vertebrate and insect species—2% and 3%, respectively. According to these criteria, one might accept that all *Ma. flaveola* sequences belong to a single species. However, if the *Ma. flaveola* G I sequence (Amazonian) is analyzed separately, the mean pairwise distances decrease to 0.83% ± 0.20%, a value that is almost one third of the previous one and approaches those obtained for *Ma. amazonensis*, *Ma. humeralis*, and *Ma. iguassuensis* MOTUs (Table 1 and Table 4). Scarpassa et al. [38] found some divergence between sequences of supposed *Ma. flaveola* from Argentina and Puerto Rico averaging 4.60% ± 0.01% but classified only as “hidden genetic variation”. As the mean pairwise distance between the *Ma. flaveola* GI and *Ma. flaveola* GII was 9.76% ± 1.23% (Table 2), an expected value for pairs of congeneric Diptera species [90], we believe it is reasonable to suspect that they are different species, mainly in view of the strong geographic barriers separating their populations. In that case, it is more plausible to believe that the authentic, or stricto sensu (s.s.), *Ma. flaveola* is represented by lineage G II, whose member is topotypical for the species [91]. A more comprehensive sampling is necessary to verify this hypothesis.

The best ASAP partition and two others generated by ABGD considered the three *Ma. pseudotitillans* sequences analyzed as members of a single MOTU (Supplementary Table S6) with pairwise NJ distance ranging from 0.15% ± 0.15% to 3.29% ± 0.70% (Supplementary Table S5). In contrast, the RESL and mPTP algorithms equally divided the sequences into two MOTUs, *Ma. pseudotitillans* G I and *Ma. pseudotitillans* G II. According to the NJ and RESL analyses, the mean divergence between these sequence subsets is >3% (Table 2 and Table 4). According to the criteria proposed by Hebert et al. [30], these findings lead to an ambiguity that prevents the delimitation of a clear species boundary. Even though the very small sample size may eventually explain the inconclusive finding by itself, it is important to emphasize that *Ma. pseudotitillans* is present in a much larger area than that of the eastern Amazon, where the three specimens under analysis were collected. There are records of this species in different biomes in Brazil [92] and in various other South American and Caribbean countries [93]. Thus, future sampling efforts aimed at a better understanding of *Ma. pseudotitillans* taxonomy should seek to cover a wider geographic and ecosystem range.

When all 116 sequences assigned to *Ma. titillans COI* were treated as a single hypothetical taxonomic unit, the pairwise distances averaged 4.39% ± 0.54% (Table 1), exceeding the 3% limit proposed for insects [30]. In fact, two MOTUs, namely, *Ma. titillans* G I and *Ma. titillans* G II, were generated when sequences were analyzed regardless of the clustering method (Table 3). When the distance analysis was performed separately for these two MOTUs using MEGA X, pairwise distances for *Ma. titillans* G I and *Ma. titillans* G II averaged 0.49% ± 0.11% and 0.31% ± 0.11%, respectively, while the inter-MOTU mean pairwise distance was 10.65% ± 1.34%. These genetic distance results were congruent with those obtained from RESL (Table 4). By assuming that an average interspecific genetic distance 10 times greater than the mean intraspecific distances indicates the existence of species complexes [94], such results may strongly support the hypothesis of a species complex in a henceforth lato sensu (l.s.) *Ma. titillans*. Only *Ma. titillans* G I included sequences from specimens collected in a location geographically close to the type locality, which therefore apparently indicates *Ma. titillans* s.s. Differently represented by specimens from southeastern Brazil, *Ma. titillans* G II is likely to be an unknown species. This hypothesis is supported by differences we found in the male genitalia morphology of *Ma. titillans* G I (Supplementary Figure S2a,b) and *Ma. titillans* G II (Supplementary Figure S2c,d). The main perceived differences were related to the shape of the dorsal margin, basolateral lobe, gonostillar claw, and contour of the aedeagus. In the early 1970s, variations observed in morphological characters of immature *Ma. titillans* had already led to the hypothesis that it was a species complex [66].

The average of the pairwise K2p genetic distances computed by the NJ analysis with all sequences morphologically assigned to *Ma. indubitans* (5.74% ± 0.57%, Table 1) also exceeded the 3% intraspecific threshold [30]. By separating them according to the MOTUs defined in the topology of the NJ tree, the mean intra-MOTU distances for *Ma. indubitans* G II and *Ma. indubitans* G III were 0.20% ± 0.14% and 0.32% ± 0.08% (Table 1), respectively, while the inter-MOTU averaged 13.04% ± 1.41 (Table 2). Aggregated as a single MOTU, as suggested by the ABGD result under *P* = 5.99% (Supplementary Table S6), the *Ma. indubitans* G I and *Ma indubitans* G II sequences diverged by an average of 2.12% ± 0.41% (Table 2). This last genetic distance could justify the conspecificity inference [30]. However, the single *Ma. indubitans* G I sequence was about 20 times more divergent from all *Ma indubitans* G II sequences than those seen for any pair of the latter (Supplementary Table S5). Such set of genetic distances can generate some controversy, even more so with sympatric populations (*Ma. indubitans* haplotypes 1 and 4, in Supplementary Table S1). Therefore, it is prudent to increase the sampling from which *Ma. indubitans* G I and *Ma. indubitans* G II specimens are obtained for reanalysis before concluding whether they are part of one or more species.

None of the 70 individual sequences of *Ma. indubitans* G III (15 haplotypes) diverged less than 11.47% ± 1.33% from any other in *Ma. indubitans* G I and *Ma. indubitans* G II (Supplementary Table S5). Among those 70 specimens from which the sequences were extracted and whose genetic distances averaged 0.32% ± 0.08% (Table 1), 22 were topotypes collected from the metropolitan area of Belém in the state of Pará (Supplementary Table S1). Therefore, it seems appropriate to assume that *Ma. indubitans* is a species complex that comprises at least two isomorphic species—*Ma. indubitans* G I and *Ma. indubitans* G II—and *Ma. indubitans* s.s. (*Ma. indubitans* G III).

Neighbor joining and all other clustering methods unexpectedly assembled the supposed *Ma. indubitans* GenBank sequences from Colombia (MN997669–MN997672) and the *Ma. dyari* BOLD sequence from Puerto Rico in a MOTU named *Ma. dyari*/*indubitans*. Scarpassa et al. [38] obtained the same finding when they used NJ analysis and Bayesian inference sequences to compare *Ma. dyari* from North, Central, and South America and sequences from Colombia assigned to *Ma. indubitans*, all downloaded from GenBank. For *Ma. dyari*/*indubitans*, the intra-MOTU genetic K2p distances averaged 0.37% ± 0.17% (Table 1 and Table 4) and its NN—*Ma. indubitans* G III—diverged by more than 15 times (Table 2 and Table 4). *Ma. dyari,* morphologically very similar to *Ma. indubitans*, was described in samples collected from Jamaica [66], and its known distribution extends from the southern United States to Colombia [93]. Thus, it is reasonable to believe that the Colombian specimens assigned to *Ma. indubitans* we analyzed are actually *Ma. dyari*, which would explain the relatively high genetic divergence in relation to our *Ma. indubitans* sequences.

In addition, when comparing our findings to those of Scarpassa et al. [38], there is an important disagreement to highlight. The authors inferred that certain *Mansonia* (*Mansonia*) specimens they sampled in Porto Velho in the state of Rondônia belonged to a cryptic species more closely related to *Ma. dyari* and provisionally named “*Ma*. near *dyari*”. However, we also assembled collections in Porto Velho during practically the same periods and believe that the “*Ma*. near *dyari*” of Scarpassa et al. [38] may actually be *Ma. indubitans* s.s., because: (i) there is no previous record of *Ma. dyari* in Brazil; (ii) adult females of *Ma. indubitans* can be easily misidentified as *Ma. dyari* because of the high degree of morphological similarity between these species; (iii) 21.5% of the mosquitoes we sampled in Porto Velho were morphologically assigned to *Ma. indubitans*, while Scarpassa et al. [38] recorded only 2% of the species in their sample; (iv) the mean genetic K2p distance reported by Scarpassa et al. [38] for the “*Ma.* near *dyari*” DNA barcode sequences (0.10% ± 0.10%) was compatible with the mean intra-MOTU divergence we found for *Ma. indubitans* G III DNA barcode sequences (0.32% ± 0.08%, Table 1), some of which are topotypical; and (v) Scarpassa et al.’s. “*Ma.* near *dyari*” and “original” *Ma*. *dyari* are mutually NN [38] (mean K2p distance = 6.6% ± 1.70%), as well as our *Ma. indubitans* G III and *Ma. dyari*/*indubitans* (mean K2p distance = 6.42% ± 0.96%) (Table 2).

*Ma. fonsecai* was primarily described by Pinto [95] in specimens collected from eastern Bolivia, but shortly afterwards, the classification changed to *Ma. indubitans* synonymy [63]. About 70 years later, Barbosa et al. [68] revalidated *Ma. fonsecai* after morphologically comparing its holotype and paratypes with mosquito samples from southern Brazil. It has been suspected that different species could have been misidentified as *Ma. indubitans* in South America because of morphological variations in the gonostylus detected in a priori *Ma. indubitans* populations from Colombia [66]. We also found morphological variations in the gonostylus of males morphologically assumed as *Ma. fonsecai*. This was more conspicuous when comparing the gonostylus of *Ma. fonsecai* G I and *Ma. fonsecai* G III (Supplementary Figure S3). These morphological variations were congruent with the genetic divergences among the *Ma. fonsecai* related MOTUs.

The genetic K2p distance among the seven haplotypes of the specimens morphologically assigned to *Ma. fonsecai* ranged from 0.15% ± 0.15% to 12.39% ± 1.42% (Supplementary Table S5). Haplotypes 1 and 7 were quite divergent among themselves and in relation to the others (≥9.93% ± 1.26%, Supplementary Table S5). This result corroborates their isolations into two MOTUs—*Ma. fonsecai* G III and *Ma. fonsecai* G IV, respectively—according to all automated clustering methods (Table 3). The hypothesis that *Ma. fonsecai* is a species complex becomes even more plausible given that the *Ma. fonsecai* G III and *Ma. fonsecai* G IV populations are separated from that explored in Curitiba for *Ma. fonsecai* revalidation [68] by approximately 160 km and the Serra do Mar, a mountain range with peaks that rise over 1800 m above sea level [96]. It is more likely that the *Ma*. *fonsecai* s.s. is represented here by specimens linked to MOTUs *Ma*. *fonsecai* G I and/or *Ma*. *fonsecai* G II, all of which practically originate from Curitiba. The only exception is a single *Ma*. *fonsecai* G II specimen from the state of São Paulo (Supplementary Table S1).

Five remaining *Ma. fonsecai* haplotypes were grouped into a single MOTU by most clustering methods (Table 3), including mPTP, which, unlike ABGD, ASAP and RESL, is based on phylogenetic assumptions [84]. Only RESL separated these sequences into two different MOTUs, *Ma. fonsecai* G I and *Ma. fonsecai* G II, mutually NN to each other (K2p distance = 1.83%). Their mean inter-MOTU pairwise K2p genetic distance was less than 10 times higher than the *Ma. fonsecai* G I mean intra-MOTU divergence (Table 1, Table 2 and Table 4), discrediting the hypothesis that they are two species [94]. Moreover, the average of pairwise distances computed for the above-mentioned *Ma. fonsecai* G I + G II single MOTU (1.39% ± 0.35%, Table 1) was lower than the 3% threshold for insect intraspecific divergence [30]. Two examples from the literature based on K2p distances corroborate the hypothesis generated by the ABGD, ASAP, and mPTP analyses, according to which *Ma. fonsecai* G I and *Ma. fonsecai* G II are most likely the same species. Cywinska et al. [45] sampled 37 mosquito species in Canada and found that 98% of the conspecific sequences diverged <2% from each other, although Beebe [26] suggested that the small divergence may be related to possible sampling limitations. Similarly, Wang et al. [57] analyzed 122 mosquito species from China and reported that about 98% of the conspecific sequences diverged ≤1.67%, while more than 98% of interspecific divergences ranged from 2.3% to 21.8%.

Despite the evidence for conspecificity of *Ma. fonsecai* G I and *Ma. fonsecai* G II, some caution is still necessary regarding this issue. This is so because there may be genetic exchange among lineages that have diverged abruptly or recently, obscuring their relationships [97]. In principle, by improving the sampling design and/or using additional molecular markers, it is possible to overcome controversies regarding the barcode gap [26]. Nevertheless, the *Ma*. *fonsecai* s.s. is more likely represented here by specimens linked to MOTUs *Ma*. *fonsecai* G I and/or *Ma*. *fonsecai* G II, all of which practically originate from Curitiba. The only exception is a single *Ma*. *fonsecai* G II specimen from the state of São Paulo (Supplementary Table S1).

Ultimately, no overlap was observed between maximum intra-MOTU and minimum inter-MOTU K2p genetic distances, which ranged from about four to >20 times the former. The existence of clear barcode gaps [86,98] means that DNA barcodes are useful to distinguish congeneric species [99]. In addition, the ratio of the number of BINs generated by RESL (Table 4) to the number of recognized morphospecies (BIN/SP ≅ 2) indicates that there are species neglected by the current taxonomic system, as is ordinary for poorly known taxa [100] such as *Mansonia* (*Mansonia*). As isomorphic unrecognized species gathered into single morphological taxa may differ in pathogen transmission potentials [101], these findings deserve attention.

## 5. Conclusions

The results of the analyses carried out with DNA barcode sequences made it possible to unequivocally distinguish all eight initially listed *Mansonia* (*Mansonia*) morphospecies from each other. Unprecedented records of DNA barcode sequences assigned to *Ma. fonsecai*, *Ma. iguassuensis*, and *Ma. pseudotitillans* were presented. *Ma. amazonensis*, *Ma. flaveola*, *Ma. humeralis*, *Ma. indubitans*, and *Ma. titillans* sequences substantially complement the DNA barcode library for *Mansonia* (*Mansonia*) spp., providing greater reliability to its molecular specific identification [29,70].

The molecular evidence gathered here suggests the existence of isomorphic species complexes related to *Ma. flaveola*, *Ma. fonsecai*, *Ma. indubitans*, *Ma. pseudotitillans*, and *Ma. titillans*. However, these findings should be treated as preliminary for the delimitation of *Mansonia* (*Mansonia*) species [82]. Regardless of reformulation of sampling strategies, it is recommended that new species hypotheses be tested through the association of other molecular markers [102]. Beebe [26] provides an online supplementary table that compiles several molecular markers from the literature applied to the barcoding of mosquito genera.

## Figures and Tables

**Table 1:** Average of intra-MOTU K2p distances computed by MEGA X with 338 *Mansonia* (*Mansonia*) spp. DNA barcode sequences (11 of them from GenBank and BOLD). The number of sequences (n) and corresponding haplotypes (h) per MOTU are given. In the second and third columns, the samples morphologically identified as *Ma. flaveola*, *Ma. fonsecai*, *Ma. indubitans*, *Ma. pseudotitillans*, and *Ma. titillans* were subdivided into groups corresponding to the MOTUs shown in the NJ tree topology. It was not possible to calculate the average of distances for MOTUs composed of only one haplotype. Asterisks show the highest value for each column.

MOTUs	n	h	Intra-MOTU K2p Distances (%)		
*Ma. amazonensis*	24	17	0.54 ± 0.13	0.54 ± 0.13	0.54 ± 0.13
*Ma. flaveola* G I	13	11	2.32 ± 0.29	0.83 ± 0.20	0.83 ± 0.20 *
*Ma. flaveola* G II	1	1		–	–
*Ma. fonsecai* G I	7	3	6.52 ± 0.65 *	1.39 ± 0.33	0.30 ± 0.17
*Ma. fonsecai* G II	4	2			0.15 ± 0.15
*Ma. fonsecai* G III	16	1		–	–
*Ma. fonsecai* G IV	1	1		–	–
*Ma. humeralis*	64	17	0.43 ± 0.13	0.43 ± 0.13	0.43 ± 0.13
*Ma. iguassuensis*	5	4	0.36 ± 0.18	0.36 ± 0.18	0.36 ± 0.18
*Ma. indubitans* G I	1	1	5.74 ± 0.57	2.12 ± 0.41 *	–
*Ma. indubitans* G II	8	3			0.20 ± 0.14
*Ma. indubitans* G III	70	15		0.32 ± 0.08	0.32 ± 0.08
*Ma. dyari/indubitans*	5	5		0.37 ± 0.17	0.37 ± 0.17
*Ma. pseudotitillans* G I	2	2	2.19 ± 0.46	0.15 ± 0.15	0.15 ± 0.15
*Ma. pseudotitillans* G II	1	1		–	–
*Ma. titillans* G I	87	30	4.39 ± 0.54	0.49 ± 0.11	0.49 ± 0.11
*Ma. titillans* G II	29	10		0.31 ± 0.11	0.31 ± 0.11

**Table 2:** Average of inter-MOTU K2p distances (lower left) and respective standard errors (upper right) computed by MEGA X with 338 *Mansonia* (*Mansonia*) spp. DNA barcode sequences (11 of them from GenBank and BOLD). Asterisk shows the lowest value.

Lineages		Between Lineages K2p Distances and Standard Errors (%)																
		1	2	3	4	5	6	7	8	9	10	11	12	13	14	15	16	17
1	*Ma. dyari/indubitans*		1.41	1.70	1.61	1.51	1.47	1.38	1.28	1.30	1.47	0.96	1.31	1.28	1.39	1.30	1.50	1.33
2	*Ma. humeralis*	12.95		1.48	1.44	1.39	1.52	1.67	1.41	1.62	1.63	1.57	1.51	1.52	1.60	1.41	1.59	1.57
3	*Ma. flaveola* G I	16.52	13.55		1.23	1.63	1.73	1.84	1.69	1.86	1.95	1.78	1.63	1.66	1.68	1.72	1.88	1.79
4	*Ma. flaveola* G II	14.77	12.95	9.76		1.41	1.51	1.72	1.57	1.70	1.74	1.57	1.60	1.63	1.68	1.57	1.89	1.74
5	*Ma. pseudotitillans* G I	13.68	12.42	15.73	11.56		0.70	1.48	1.48	1.51	1.57	1.69	1.40	1.32	1.40	1.49	1.69	1.44
6	*Ma. pseudotitillans* G II	13.70	13.77	16.15	12.55	3.21		1.49	1.51	1.52	1.60	1.63	1.44	1.30	1.40	1.53	1.75	1.53
7	*Ma. amazonensis*	12.42	15.10	18.44	16.20	12.69	12.68		1.32	1.42	1.52	1.57	1.54	1.57	1.62	1.49	1.64	1.67
8	*Ma. iguassuensis*	10.39	12.37	16.18	15.10	13.45	14.10	10.95		1.42	1.46	1.47	1.62	1.53	1.61	1.38	1.58	1.40
9	*Ma. indubitans* G II	11.66	16.17	18.63	16.16	14.21	14.85	13.52	12.68		0.77	1.41	1.53	1.44	1.55	1.40	1.61	1.56
10	*Ma. indubitans* G I	13.61	16.43	19.44	16.42	15.01	15.66	15.35	13.28	4.03		1.60	1.51	1.52	1.60	1.45	1.58	1.67
11	*Ma. indubitans* G III	6.42	14.87	16.74	14.36	15.74	15.63	13.67	12.21	13.04	14.91		1.47	1.40	1.49	1.48	1.66	1.40
12	*Ma. fonsecai* G III	11.69	14.58	16.11	14.92	13.55	13.84	14.10	15.48	12.85	13.82	13.47		1.31	1.36	1.33	1.53	1.40
13	*Ma. fonsecai* G II	10.96	14.38	16.31	15.48	12.01	12.10	13.62	13.91	11.42	13.09	11.74	10.02		0.54	1.36	1.63	1.48
14	*Ma. fonsecai* G I	12.31	15.79	16.60	15.99	13.18	13.09	14.37	14.81	13.17	13.96	13.09	11.00	2.15 *		1.42	1.68	1.57
15	*Ma. fonsecai* G IV	10.84	13.12	16.55	15.48	14.10	14.93	13.31	12.11	11.78	12.74	12.92	11.14	11.22	12.09		1.59	1.42
16	*Ma. titillans* G I	12.20	15.53	19.43	18.43	15.80	16.34	15.18	14.75	14.14	14.97	13.91	13.45	13.63	14.69	13.40		1.34
17	*Ma. titillans* G II	10.31	14.77	17.03	15.95	13.42	14.23	15.14	12.39	13.68	15.14	12.14	11.99	12.71	13.54	10.98	10.65	

**Table 3:** Comparison of automated clustering methods based on genetic K2p distances (ABGD, ASAP, and RESL) and number of substitutions (mPTP) with 338 *Mansonia* (*Mansonia*) spp. DNA barcode sequences (11 of them from GenBank and BOLD). The dotted lines delimit the MOTUs generated by the different clustering methods (columns).

Cluster Analyses Results			
ABGD * (n)	ASAP ** (n)	RESL (n)	mPTP (n)
*Ma. amazonensis* (24)	*Ma. amazonensis* (24)	*Ma. amazonensis* (24)	*Ma. amazonensis* (24)
*Ma. flaveola* G I (13)	*Ma. flaveola* G I (13)	*Ma. flaveola* G I (13)	*Ma. flaveola* G I (13)
*Ma. flaveola* G II (1)	*Ma. flaveola* G II (1)	*Ma. flaveola* G II (1)	*Ma. flaveola* G II (1)
*Ma. fonsecai* G I + G II (11)	*Ma. fonsecai* G I + G II (11)	*Ma. fonsecai* G I (7)	*Ma. fonsecai* G I + G II (11)
		*Ma. fonsecai* G II (4)	
*Ma. fonsecai* G III (16)	*Ma. fonsecai* G III (16)	*Ma. fonsecai* G III (16)	*Ma. fonsecai* G III (16)
*Ma. fonsecai* G IV (1)	*Ma. fonsecai* G IV (1)	*Ma. fonsecai* G IV (1)	*Ma. fonsecai* G IV (1)
*Ma. humeralis* (64)	*Ma. humeralis* (64)	*Ma. humeralis* (64)	*Ma. humeralis* (64)
*Ma. iguassuensis* (5)	*Ma. iguassuensis* (5)	*Ma. iguassuensis* (5)	*Ma. iguassuensis* (5)
*Ma indubitans* G I (1)	*Ma indubitans* G I (1)	*Ma indubitans* G I (1)	*Ma indubitans* G I (1)
*Ma indubitans* G II (8)	*Ma indubitans* G II (8)	*Ma indubitans* G II (8)	*Ma indubitans* G II (8)
*Ma indubitans* G III (70)	*Ma indubitans* G III (70)	*Ma indubitans* G III (70)	*Ma indubitans* G III (70)
*Ma. dyari/indubitans* (5)	*Ma. dyari/indubitans* (5)	*Ma. dyari/indubitans* (5)	*Ma. dyari/indubitans* (5)
*Ma. pseudotitillans* G I + G II (3)	*Ma. pseudotitillans* G I + G II (3)	*Ma. pseudotitillans* G I (2)	*Ma. pseudotitillans* G I (2)
		*Ma. pseudotitillans* G II (1)	*Ma. pseudotitillans* G II (1)
*Ma. titillans* G I (87)	*Ma. titillans* G I (87)	*Ma. titillans* G I (87)	*Ma. titillans* G I (87)
*Ma. titillans* G II (29)	*Ma. titillans* G II (29)	*Ma. titillans* G II (29)	*Ma. titillans* G II (29)

* *P* = 2.15–3.59%. ** *dT* = 3.50–10.09%.

**Table 4:** Mean and maximum (Max.) intra-MOTU and nearest neighbor (NN) K2p distances generated by the RESL algorithm implemented in BOLD Systems workbench with 338 *Mansonia* (*Mansonia*) spp. DNA barcode sequences (11 of them from GenBank and BOLD).

MOTUs	n	Intra-MOTU K2p Distances (%)		NN Distances (%)
		Mean	Max.	
*Ma. humeralis*	64	0.27	0.76	11.16
*Ma. flaveola* G I	13	0.83	1.68	8.72
*Ma. amazonensis*	24	0.44	0.92	9.79
*Ma. titillans* G I	87	0.25	1.07	9.33
*Ma. indubitans* G III	70	0.14	0.61	5.81
*Ma. indubitans* G II	8	0.10	0.31	3.82
*Ma. indubitans* G I	1	–	–	3.82
*Ma. titillans* G II	29	0.17	0.46	9.33
*Ma. pseudotitillans* G II	1	–	–	3.06
*Ma. pseudotitillans* G I	2	0.15	0.15	3.06
*Ma. iguassuensis*	5	0.34	0.46	9.48
*Ma. fonsecai* G III	16	0.00	0.00	9.33
*Ma. fonsecai* G IV	1	–	–	9.94
*Ma. dyari/indubitans*	5	0.37	0.61	5.81
*Ma. fonsecai* G II	4	0.08	0.15	1.83
*Ma. flaveola* G II	1	–	–	8.72
*Ma. fonsecai* G I	7	0.22	0.46	1.83

## Data Availability

Not applicable.

## Supplementary Materials

The following supporting information can be downloaded at: https://www.mdpi.com/article/10.3390/genes14061127/s1. Figure S1: NJ topology estimated by MEGA X using K2p genetic distances (scale bar) with 124 *Mansonia* (*Mansonia*) spp. *COI* haplotype sequences, eight of which represent sequences extracted from GenBank and BOLD (asterisk). The tree is rooted on the *Ps. longipalpus* sequence, which, similar to those of the other outgroup members, is highlighted in red. Bootstrap values (1000 replicates) are shown above or to the left of the branches; Figure S2: Morphological differences between gonostylus and aedeagus of *Ma. titillans* G I (a and b, respectively) and *Ma. titillans* G II (c and d, respectively); Figure S3: Morphological differences between gonostylus of Ma. fonsecai G I (a) and Ma. fonsecai G III (b); Table S1: Additional individual data on 327 *Mansonia* (*Mansonia*) spp. specimens from which new DNA barcode sequences were extracted for the study and another 11 sequences downloaded from GenBank or BOLD. Adult collections made by active search, human attraction, barrier screen method, and Shannon light trap were supported by electric catchers. Dashes indicate absence of specific data; Table S2: Similarities between DNA barcode sequences of *Mansonia* spp. from GenBank database and 327 *Mansonia* (*Mansonia*) spp. sample; Table S3: Average of inter-MOTU Jukes–Cantor distances (lower left) and respective standard errors (upper right) computed by MEGA X with 338 *Mansonia* (*Mansonia*) spp. DNA barcode sequences (11 of them from GenBank and BOLD). Asterisk shows the lowest value; Table S4: Average of inter-MOTU Tamura three-parameter distances (lower left) and respective standard errors (upper right) computed by MEGA X with 338 *Mansonia* (*Mansonia*) spp. DNA barcode sequences (11 of them from GenBank and BOLD). Asterisk shows the lowest value; Table S5: All pairwise K2p distances (lower left) and standard errors (upper right) computed by MEGA X for 124 *Mansonia* (*Mansonia*) spp. haplotype sequences; Table S6: Comparison of all automated clustering methods results based on genetic K2p distances (ABGD, ASAP and RESL) and number of substitutions (mPTP) with 338 *Mansonia* (*Mansonia*) spp. DNA barcode sequences (11 of which from GenBank and BOLD). The rows delimit the MOTUs generated by the different clustering methods (columns). The partitions of ABGD and ASAP are shown for different values of prior limit to intraspecific diversity (*P*) and threshold distance (*d_T_*).
