# Supplementary material for: DNA Barcodes of Mansonia (Mansonia) Blanchard, 1901 (Diptera, Culicidae)
Source: Genes (Basel). 2023 May 23;14(6):1127. doi: 10.3390/genes14061127 (PMC10298705; doi:10.3390/genes14061127)
Supplement: Supplementary file 1 [file genes-14-01127-s001.zip › Figure S1.pdf]

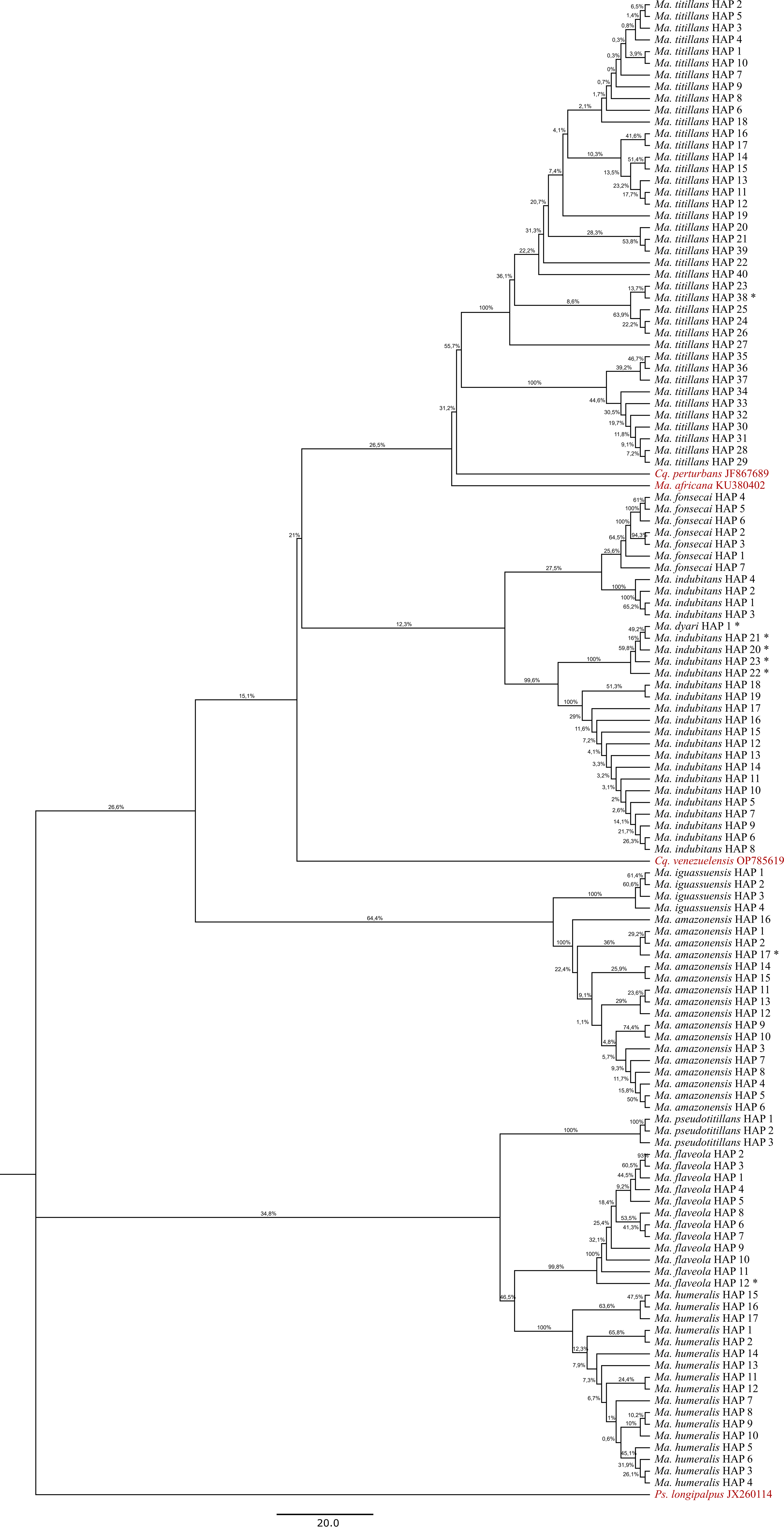

Figure S1. NJ topology estimated by MEGA X using K2p genetic distances (scale bar) with 124 *Mansonia* (*Mansonia*) spp. COI haplotype sequences, eight of which represent sequnces extracted from GenBank and BOLD (asterisk). The tree is rooted on *Ps. longipalpus* sequence, which, like those of other outgroup members, is highlighted in red. Bootstrap values (1,000 replicates) are shown above or to the left of the branches.
