# Supplementary figures and images for: DNA Barcodes of Mansonia (Mansonia) Blanchard, 1901 (Diptera, Culicidae)
Source: Genes (Basel). 2023 May 23;14(6):1127. doi: 10.3390/genes14061127 (PMC10298705; doi:10.3390/genes14061127)

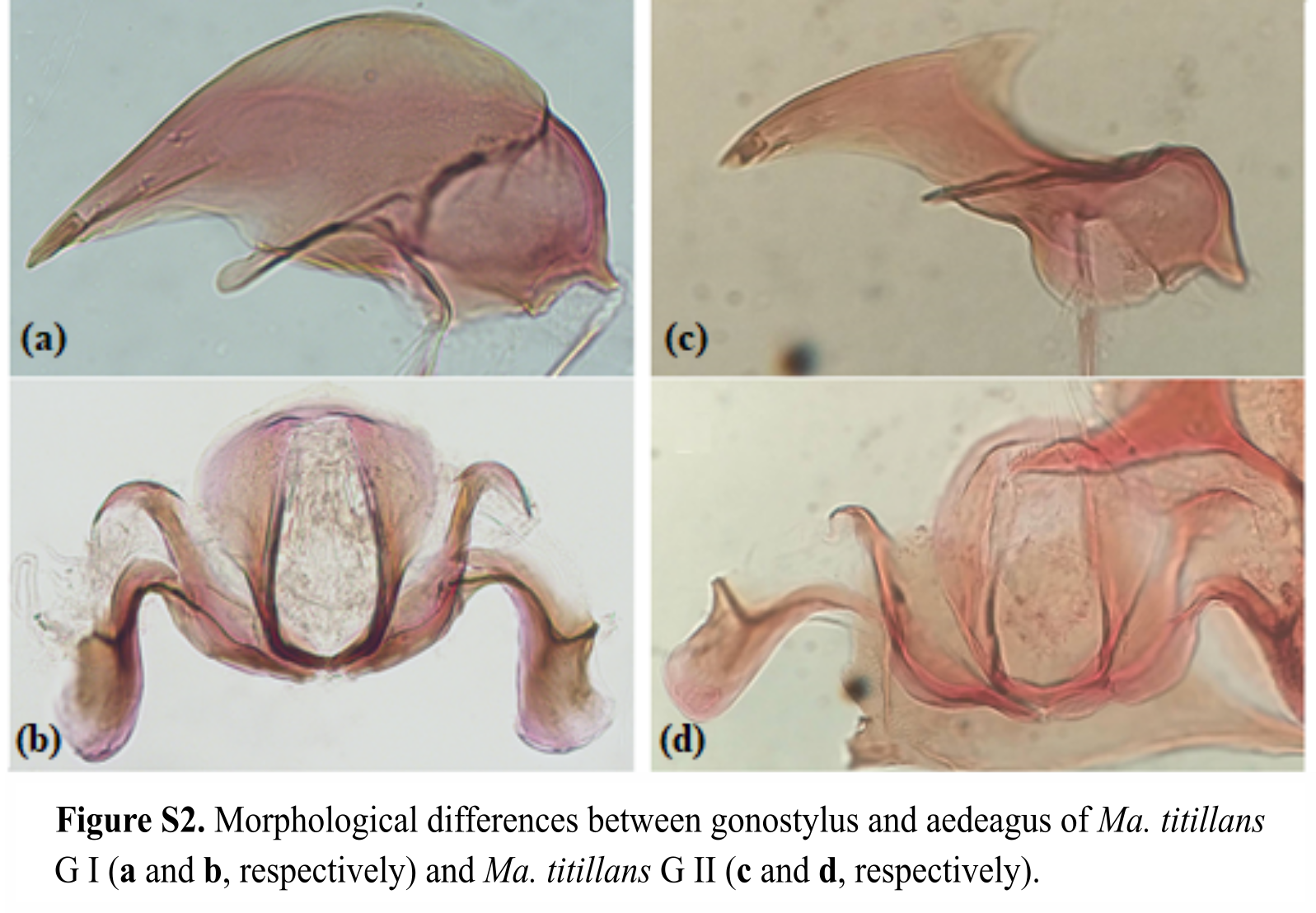

Supplement: Supplementary file 1 [file genes-14-01127-s001.zip › Figure S2.png]

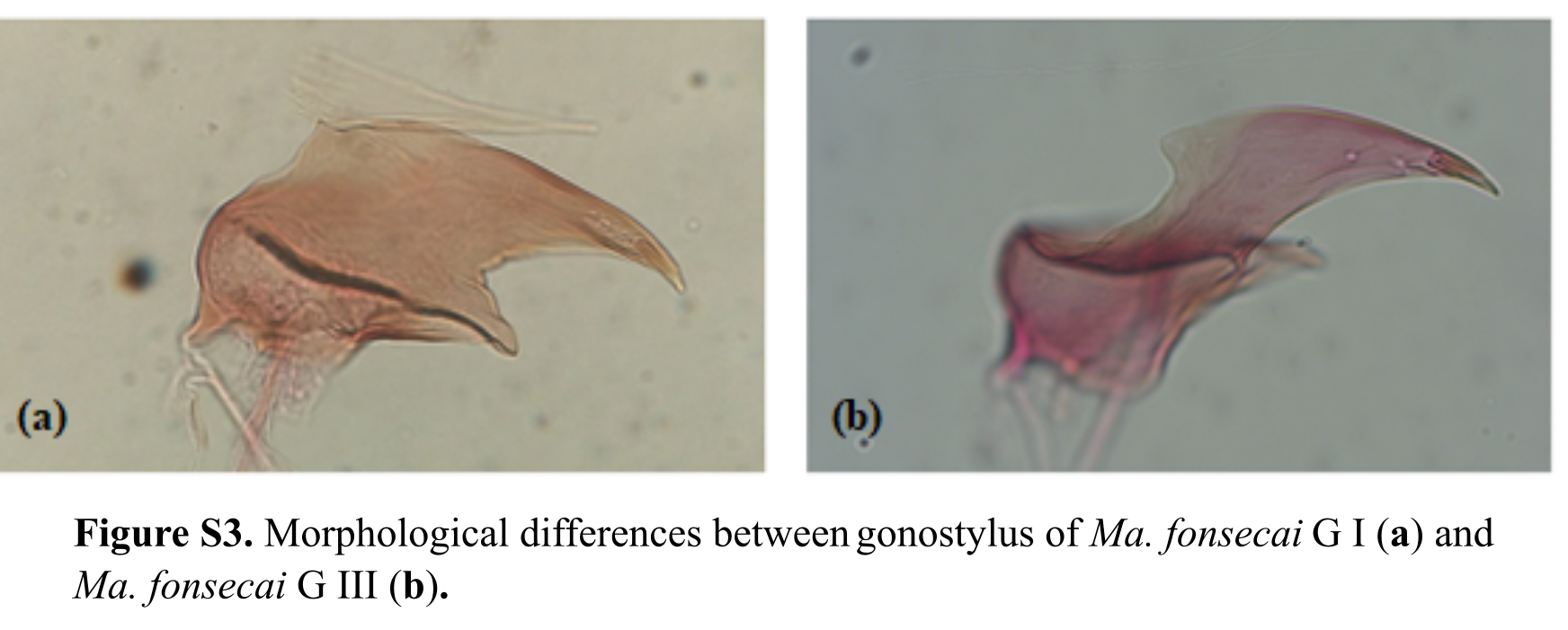

Supplement: Supplementary file 1 [file genes-14-01127-s001.zip › Figure S3.png]
